# RETRACTED: Liu et al. Prediction of Ovarian Cancer Response to Therapy Based on Deep Learning Analysis of Histopathology Images. *Cancers* 2023, *15*, 4044

**DOI:** 10.3390/cancers16030493

**Published:** 2024-01-24

**Authors:** Yuexin Liu, Barrett C. Lawson, Xuelin Huang, Bradley M. Broom, John N. Weinstein

**Affiliations:** 1 Department of Bioinformatics and Computational Biology, The University of Texas MD Anderson Cancer Center, Houston, TX 77030, USA; 2 Department of Pathology, The University of Texas MD Anderson Cancer Center, Houston, TX 77030, USA; 3 Department of Biostatistics, The University of Texas MD Anderson Cancer Center, Houston, TX 77030, USA; 4 Department of Systems Biology, The University of Texas MD Anderson Cancer Center, Houston, TX 77030, USA

The journal and authors wish to retract the article entitled ‘Prediction of Ovarian Cancer Response to Therapy Based on Deep Learning Analysis of Histopathology Images’ cited above [1].

Soon after publication, the authors contacted the Editorial Office to request retraction of the article [1]. Their further extensive computations and deeper exploration of the learning process had revealed an error, and it was found that the results presented were, therefore, statistical outliers for reasons that apparently related to the random selection of held-out test data. As a result, the original findings of clear prediction by the deep learning model could not be validated.

Adhering to our complaint’s procedure, an investigation was conducted by the Editorial Office and Editorial Board that confirmed that the central finding cannot be considered to be reliable. Consequently, the Editorial Office, the Editorial Board, and the authors have decided to retract the article [1] as per MDPI’s retraction policy (https://www.mdpi.com/ethics#_bookmark30).

This retraction was approved by the Editor-in-Chief of the journal *Cancers*.

The authors agreed to this retraction.
